# ASO Author Reflections: The Role of Established Prognostic Factors in Long-Term Survival After Resection of Pancreatic Ductal Adenocarcinoma

**DOI:** 10.1245/s10434-024-15391-w

**Published:** 2024-05-20

**Authors:** Omar Mahmud, Ammar A. Javed, Asad Saulat Fatimi, Alyssar Habib, Mahip Grewal, Jin He, Christopher L. Wolfgang, Marc G. Besselink, PANC-PALS consortium

**Affiliations:** 1 NYU Langone Health, NYU Grossman School of Medicine, New York City, NY USA; 2 Medical College, Aga Khan University, Karachi, Pakistan; 3 Amsterdam UMC, Department of Surgery, University of Amsterdam, Amsterdam, The Netherlands; 4 Cancer Center Amsterdam, Amsterdam, The Netherlands; 5 Department of Surgery, Johns Hopkins School of Medicine, Baltimore, MD USA

## Past

The prognosis of patients with pancreatic ductal adenocarcinoma (PDAC) remains dismal despite improvements in surgical techniques and the introduction of multiagent systemic therapies.^1,2^ Although the rate of long-term survival (LTS, > 5 years) in PDAC is increasing, the rarity of these patients remains a challenge in most studies.^1,2^ Furthermore, prior studies on true LTS are mostly based on survival estimates, rather than complete follow-up, and typically consist of small single-center cohorts.^3^

## Present

The systematic review included 27,091 patients with resected PDAC, all from studies reporting true LTS (i.e., with complete follow-up).^4^ The median rate of LTS was 18% (interquartile range 13–21%) across studies from North America, Europe, and Asia. Pooled associations between established clinicopathologic factors and LTS included lower grade of tumor differentiation [odds ratio (OR) 1.75], lower American Joint Commission on Cancer (AJCC) stage (OR 3.4), receipt of adjuvant chemotherapy (OR 1.75), and negative surgical margins (OR 2.4). Patients with LTS had lower odds of having features of aggressive tumor biology, such as perineural (OR 0.46), lymphatic (OR 0.44), or vascular (OR 0.52) invasion. Most pooled associations had modest quantitative effect sizes. The largest association between LTS and clinicopathological characteristics was observed for localized disease (OR 6.3), but even the presence of distant metastases did not preclude LTS. Limited data with mixed results were seen in regard to the association of neoadjuvant therapy with LTS.

## Future

This review provides pooled associations between established clinicopathologic factors and true LTS in patients with resected PDAC. The findings in the present study will act as a baseline for future multicenter studies, which are needed to further study and characterize LTS, especially in the neoadjuvant era. Our results also highlight the fact that no single factor, or combination of factors, can accurately explain why some patients achieve LTS in resected PDAC while others do not. Novel biomarkers that are representative of tumor and host biology need to be studied to improve prognostication and accurate prediction of long-term survival.
